# Advancements in multi-omics research to address challenges in Alzheimer’s disease: a systems biology approach utilizing molecular biomarkers and innovative strategies

**DOI:** 10.3389/fnagi.2025.1591796

**Published:** 2025-07-23

**Authors:** Madison Cardillo, Keyura Katam, Prashanth Suravajhala

**Affiliations:** ^1^Department of Biological Sciences, Florida A&M University, Tallahassee, FL, United States; ^2^College of Arts and Sciences, Florida State University, Tallahassee, FL, United States; ^3^College of Liberal Arts, University of Florida, Gainesville, FL, United States; ^4^Department of Biosciences, Manipal University Jaipur, Dehmi Kalan, India; ^5^Bioclues.org, Hyderabad, India

**Keywords:** multi-omics, biomarkers, proteomics, metabolomics, genomics, CRISPR, radiomics, Alzheimer’s biomarkers, machine learning and radiomics

## Abstract

Alzheimer’s disease (AD) is a growing global challenge, representing the most common neurodegenerative disorder and affecting millions of lives. As life expectancy continues to rise and populations expand, the number of individuals coping with the cognitive declines caused by AD is projected to double in the coming years. By 2050, we may see over 115 million people diagnosed with this devastating condition. Unfortunately, while we currently lack effective cures, there are preventative measures that can slow disease progression in symptomatic patients. Thus, research has shifted toward early detection and intervention for AD in recent years. With technological advances, we are now harnessing large datasets and more efficient, minimally invasive methods for diagnosis and treatment. This review highlights critical demographic insights, health conditions that increase the risk of developing AD, and lifestyle factors in midlife that can potentially trigger its onset. Additionally, we delve into the promising role of plant-based metabolites and their sources, which may help delay the disease’s progression. The innovative multi-omics research is transforming our understanding of AD. This approach enables comprehensive data analysis from diverse cell types and biological processes, offering possible biomarkers of this disease’s mechanisms. We present the latest advancements in genomics, transcriptomics, Epigenomics, proteomics, and metabolomics, including significant progress in gene editing technologies. When combined with machine learning and artificial intelligence, multi-omics analysis becomes a powerful tool for uncovering the complexities of AD pathogenesis. We also explore current trends in the application of radiomics and machine learning, emphasizing how integrating multi-omics data can transform our approach to AD research and treatment. Together, these pioneering advancements promise to develop more effective preventive and therapeutic strategies soon.

## 1 Background

Alzheimer’s disease (AD) is primarily characterized by the dysfunction of several brain networks responsible for maintaining homeostasis and intracellular signaling. This disease poses numerous healthcare challenges, particularly the increasing prevalence of aging populations and the lack of effective curative treatments. Current therapies mainly aim to alleviate symptoms rather than target the underlying causes. Furthermore, the lack of early detection methods complicates the diagnosis. The complex pathology of AD, marked by the accumulation of amyloid plaques and tau tangles, makes developing targeted treatments challenging. The impact of AD extends beyond medical issues, affecting caregivers both financially and emotionally, thus contributing to broader societal challenges (Passeri et al., 2022). Diagnosing AD often requires years of observed cognitive decline, and this timeline can be even longer in the absence of genetic markers. A family history of Alzheimer’s often correlates with the disease’s progression, and with no cure available, early diagnosis and preventive strategies are critical to prevent irreversible brain damage. To improve our molecular understanding of AD and enhance both treatments and early diagnoses, exploring various biological processes, including genomics, Epigenomics, transcriptomics, proteomics, lipidomics, and metabolomics, is crucial. Unlike other conditions, diagnosing AD cannot be done through brain biopsy, making advancing research on cellular structures and imaging technologies vital, given the disease’s rising prevalence. Recent studies have also utilized radiomic imaging analysis and artificial intelligence (AI) to investigate cognitive impairments linked to minor vessel diseases associated with AD (Shi et al., 2020). Efforts from prevention trials and clinicians are underway to quantify and detect AD earlier and with greater accuracy through multi-omics approaches, facilitating more comprehensive analyses of neurodegenerative disorders like Alzheimer’s (Rawat et al., 2022).

## 2 History and pathogenesis

Alzheimer’s disease (AD) is a progressive neurodegenerative disorder that German psychiatrist Alois Alzheimer first identified. He observed the presence of amyloid plaques and significant neuronal loss in patients experiencing memory loss and personality changes. Later, Emil Kraepelin emphasized the severity of the disease, particularly in the cerebral cortex and medial temporal lobe, contributing to cognitive decline (Braun et al., 2022). The pathophysiology of AD involves shrinkage of the cerebral cortex and hippocampus, enlargement of the ventricles, and the presence of amyloid-beta (Aβ) plaques, as well as tau neurofibrillary tangles. Neuroinflammation arises as blood vessels age, impairing the glymphatic system and leading to the buildup of Aβ plaques. Notably, AD presents features such as granulovacuolar degeneration, which is characterized by large, double membrane-bound vacuoles in neurons (Funk et al., 2011). In 1984, the National Institute of Neurological and Communicative Disorders and Stroke (NINCDS) and the Alzheimer’s Disease and Related Disorders Association (ADRDA) established diagnostic criteria based on neuropsychological testing, progressive memory loss, and impairment in daily activities. These symptoms are most found in late-onset Alzheimer’s disease (LOAD), which usually appears after the age of 65, and early-onset Alzheimer’s disease (EOAD), which can manifest as early as a person’s 40s or 50s. Both forms often begin with mild cognitive impairment (MCI), a transitional stage between normal aging and dementia characterized by subtle cognitive deficits. While individuals with MCI can typically maintain their independence in daily life, they may experience difficulties in critical thinking, memory retention, and executive functioning. Early symptoms of MCI may include forgetting recently learned information, becoming disoriented in familiar environments, and experiencing trouble with planning or problem-solving. These impairments can be overlooked or dismissed as normal aging; however, they represent the earliest clinical indicators of Alzheimer’s disease pathology (Campbell et al., 2025). Recognizing MCI is crucial as it serves as an early marker of neurodegenerative decline and reflects the onset of underlying brain changes such as synaptic dysfunction, the accumulation of beta-amyloid plaques, and tau pathology. An increasing number of patients without cognitive or behavioral symptoms are presenting with positive biomarkers, commonly referred to as “stage 1” cases. However, in the absence of active disease progression, these findings merely indicate susceptibility to the disease (Gale, 2024). Utilizing updated criteria released in 2011, biomarkers obtained from positron emission tomography (PET) scans and cerebrospinal fluid analysis, combined with machine learning, will aid in classifying these asymptomatic disease models. Nevertheless, current diagnostic methods typically identify the disease only after it has significantly progressed and caused irreversible damage (Jack et al., 2024).

## 3 Demographics at risk

### 3.1 Age and gender

Aging is the primary risk factor for AD, and sex differences significantly affect its development and progression. Research shows that women generally have lower synapse density but higher tau and amyloid-beta (Aβ) levels than men (Sundermann et al., 2020). Key contributors to these differences include gonadal hormones and sex chromosomes. Hormones such as estrogen and testosterone influence susceptibility to the disease; estrogen plays a vital role in processes involving mitochondrial function, inflammation, glucose transport and metabolism, and cholesterol homeostasis. Both testosterone and estrogen regulate apolipoprotein E (ApoE), a key biomarker for AD (Gamache et al., 2020). Additionally, the XX chromosomes in females and the XY chromosomes in males are responsible for genetic factors affecting AD risk. For example, the loss of the Y chromosome in male AD patients can increase Aβ toxicity and lead to premature cell death (Guo et al., 2022). Postmenopausal women experience increased levels of luteinizing hormone and follicle-stimulating hormone, which may contribute to AD pathology and cognitive decline (Valencia-Olvera et al., 2023). Research has shown that estrogen can lower Aβ levels by inhibiting the production of vesicles containing amyloid precursor proteins (Arjmand et al., 2024). However, after menopause, the decline in estrogen diminishes this protective mechanism, resulting in similar metabolic conditions in both sexes (Lopez-Lee et al., 2024).

### 3.2 Cardiovascular diseases, diabetes, and other midlife risk factors

Alzheimer’s interacts with various comorbidities, such as cardiovascular disease and diabetes, which can worsen its progression. Common health problems related to AD include high cholesterol, hypertension, and diabetes. Many cases can be traced back to midlife risk factors, including smoking, elevated blood pressure, and diabetes, accounting for up to 45% of dementia cases (Malik et al., 2021). It is essential to explore these interactions to foster the development of new treatments, especially by repurposing existing medications for Alzheimer’s management. The influence of fats and proteins on brain function and dementia risk is an area that warrants additional investigation, particularly regarding AD and vascular dementia (VD). In Type 2 diabetes mellitus (T2DM), chronic hyperglycemia exacerbates amyloid beta production and tau hyperphosphorylation, which intensifies AD pathology. Impaired insulin signaling further disrupts neuronal energy metabolism, contributing to neurodegeneration in late-onset AD. Elevated blood glucose levels in T2DM can trigger the formation of advanced glycation end-products (AGEs), which promote Aβ accumulation and tau phosphorylation, leading to increased neurodegeneration.

Genetic links between dementia and conditions like hypertension and type 2 diabetes highlight the need to understand these pathways for effective prevention and treatment. Factors such as 20-Hydroxyeicosatetraenoic acid (20-HETE), which is involved in hypertension regulation and cerebral blood flow, suggest further connections with AD and stroke risks (Gonzalez-Fernandez et al., 2021). Preventing AD relies on genetic factors, cardiovascular health, and lifestyle changes, including smoking cessation and proper nutrition (Khan et al., 2023). Therefore, focusing on cardiovascular health through lifestyle modifications and nutrition is crucial for minimizing the risk of AD and related health issues.

Chronic inflammation and oxidative stress exacerbate these health conditions, and lifestyle factors like poor diet and inactivity contribute to obesity and metabolic issues impacting cognitive and cardiovascular health. While high-density lipoprotein (HDL) cholesterol is known for lowering heart disease risk, some studies have found that elevated HDL cholesterol levels may increase risks of dementia and other health problems, highlighting the need for further exploration of these associations. In older populations, metabolic syndrome (MetS) has been linked to an increased risk of cognitive decline and cardiovascular issues, emphasizing the importance of managing lipid levels to support brain health. The brain contains substantial cholesterol, essential for nerve cell function. The transport of lipoproteins, such as low-density lipoprotein (LDL) and HDL, along with apolipoproteins like ApoE, plays a crucial role in brain fat processing. The ε4 variant of ApoE is mainly associated with a heightened susceptibility to late-onset AD. Studies surrounding obesity-related dementia implicate sedentary lifestyles, chronic stress, and genetic predisposition, particularly regarding specific ApoE alleles. The relationship between cholesterol transport and cognitive decline is an evolving research area, often yielding conflicting results. Research suggests that abdominal obesity might protect cognitive health in older adults (Pereira et al., 2023). Variations in clusterin expression may impact lipid transport in the brain, structural integrity, and cognitive function. Clusterin, or apolipoprotein J (ApoJ), is a glycoprotein associated with protein folding and linked to AD, metabolic disorders, and cardiovascular diseases. At the same time, its connection to insulin resistance and dyslipidemia implies potential as a biomarker for linking AD risk with obesity-related metabolic dysfunction.

The oral microbiota may also play a role in AD progression through various mechanisms, including oxidative stress, vascular complications, neurotoxicity, and inflammation. By causing systemic inflammation, oral bacteria could disrupt the blood-brain barrier, allowing bacteria to enter the brain. Furthermore, since amyloid-β has antibacterial properties, the interactions between oral microbiota and Aβ accumulation may be significant. Oral health can also influence dementia risk by affecting sleep, physical activity, glucose metabolism, and cardiovascular health. Moreover, midlife obesity has been linked to 7.3% of AD cases, and approximately 37% of dementia patients over 65 are also diagnosed with diabetes.

Emerging evidence suggests that obstructive sleep apnea (OSA) and other sleep disorders could increase dementia risk. The exact mechanism underlying the direct relationship between OSA and elevated dementia risk remains unknown, even as numerous epidemiological studies investigate this association with cognitive decline. These factors are often assessed in more extensive cohort studies, but the precise interplay with OSA is not yet fully understood. Clarifying these connections could enhance clinical practices and dementia prevention strategies by improving risk prediction and informing personalized treatments, particularly for individuals with mild OSA. Lifestyle and overall health delay mild cognitive impairment as individuals age. Mild cognitive impairment is often seen as a precursor to AD or dementia, and research indicates that these cognitive disorders can be influenced by ethnicity. For example, some studies have found a more significant association of symptoms in Mexican Americans compared to non-Hispanic White populations (Morgenstern et al., 2024). Conversely, research involving older Black and White Brazilian communities shows minimal racial differences in the experience of these symptoms (Wilson et al., 2021). While the influence of gender is also examined, it has been determined that brain health and neuroprotection largely depend on everyday lifestyle choices and comorbidities (Mian et al., 2024).

### 3.3 Early detection and long-term effects of Alzheimer’s disease

AD’s preclinical stage can span 20–50 years before noticeable symptoms, during which Aβ and oligomer formation changes begin to affect cognitive functions. However, daily life remains largely unaffected. Individuals may notice minor cognitive declines that minimally impact daily activities as the disease progresses to the symptomatic stage. Late-onset AD typically arises after age 65, while early-onset AD is less common and often linked to hereditary factors (Li et al., 2022). Key markers of AD pathogenesis include senile plaques, synaptic loss, and neurofibrillary tangles, primarily affecting memory-related areas in the brain, such as the hippocampus and cortex. Despite advancements in identifying AD biomarkers, the disease’s exact mechanisms remain poorly understood (Monteiro et al., 2023). Noteworthy symptoms of AD’s long-term effects include memory loss and impaired judgment, complicating decision-making and task management as the disease advances. The accumulation of plaques and tangles results in irreversible brain damage, impairing cognitive function, mood, and behavior (DeTure and Dickson, 2019). At the same time, memory impairments can lead to falls and challenges in maintaining adequate nutrition and hydration (Volkert et al., 2024). These enduring effects highlight the significance of early intervention and continuous supportive care before mild to moderate symptoms of cognitive impairment begin to affect a patient’s quality of life. Utilizing biomarkers to evaluate plaques and tangles while patients remain asymptomatic may help slow the progression of AD before irreversible damage occurs (Mozersky et al., 2022).

Integrating multi-omics data with machine learning and artificial intelligence (AI) offers a deeper insight into AD pathologies. AI can simultaneously analyze the relationships between various biological components of omics studies, resulting in a comprehensive dataset model. This methodology has also proven beneficial in treating and preventing other diseases, including identifying biomarkers and developing early detection strategies. In cardiovascular diseases, advancements in multi-omics and AI utilizing RNA sequencing, whole-genome sequencing, and other classification models have achieved accurate risk predictions and efficient patient classification for further treatment (DeGroat et al., 2024). In cases of leukemia, machine learning, and deep learning methods employing multi-omics have refined unclassified datasets for predicting blood cancer outcomes. Analysis techniques used in this field include gradient boosting, logistic regression, recurrent neural networks, and feedforward neural networks. These diverse datasets, which consider patient age, sex, mutation type, treatment methods, and chromosomal data, contribute to improved care and treatment (Abbasi et al., 2024). Patients undergoing dialysis have benefited from personalized medical treatments driven by AI for kidney disease. Kidney Online program utilizes deep learning and health data to offer recipes, lifestyle interventions, early health warnings, answers to inquiries, and follow-up plans. Research indicates that this intelligent online care system effectively reduces the risk of worsening kidney disease (Liu et al., 2024). As illustrated across various diseases and health conditions, integrating these multi-omics approaches into geriatric medicine and AD research can enhance patient care through comprehensive dataset assessments. Employing new technologies like AI, stem cells, and multi-omics to bridge gaps in AD research will facilitate the creation of human models to achieve improved outcomes in personalized neuropsychiatric care (Tanaka, 2025).

## 4 Status of treatment

Treatments for AD are designed to address the underlying mechanisms of Aβ production and alleviate symptoms. Two main classes stand out among the approved pharmacological treatments: N-methyl aspartate receptor antagonists (NMDA) and cholinesterase inhibitors. NMDA receptor antagonists regulate glutamate activity, thereby preventing excitotoxicity due to excessive glutamate release, often linked to amyloid-induced neuronal damage. Meanwhile, cholinesterase inhibitors preserve acetylcholine levels by inhibiting the enzyme cholinesterase, which helps mitigate cognitive decline. Beyond pharmacotherapy, several herbal treatments exhibit the potential to influence the biological processes associated with AD.

Research into bioactive compounds from various plants highlights their promising applications in human health and natural remedy therapies. Phytochemicals are bioactive compounds found in plants that can be used in clinical applications when extracted. *Panax ginseng* is rich in compounds like gintonin, ginseng, and polysaccharides, all recognized for their health-promoting properties (Table 1). Gintonin is explicitly involved in AD management by modulating neurotransmitter levels, including acetylcholine, dopamine, and norepinephrine, promoting autophagy, and diminishing Aβ production. Natural products, including herbs and extracts, have been shown to target tau protein formation and amyloid beta effectively (Aβ) plaques due to their antioxidant properties (Lobine et al., 2021). These harmful agents arise from radical and non-radical oxygen species, reactive nitrogen species, and reactive oxygen species, which are highly chemically reactive and can contribute to oxidative damage in Alzheimer’s disease (AD), thereby impairing neuronal function. Antioxidants help mitigate this toxic stress by transforming free radicals into harmless byproducts, providing neuroprotection (Chen et al., 2021). Examples of antioxidants include *Centella Asiatica*, *Withania somnifera*, and *Crocus sativus* (Zieneldien et al., 2022). In addition to antioxidant therapies, anti-inflammatory treatments have also been found to support neuronal health. Studies in mice have demonstrated that mulberry extract can provide neuroprotection, reducing neuronal and astrocytic apoptosis. This effect is associated with an increase in anti-inflammatory cytokines (such as IL-4) and a decrease in pro-inflammatory cytokines (such as IL-1β, IL-6, and TNF-α), suggesting its potential as a therapeutic agent for neurodegenerative diseases like AD (Liu and Du, 2020). Similar studies have investigated on *Hypericum perforatum* extract and found it exhibits biological activity against Aβ-related effects (El Menuawy et al., 2024). Other anti-inflammatory herbs, such as *Scutellaria baicalensis*, *Bacopa monnieri*, and *Chlorella zofingiensis*, have also been shown to alleviate cognitive impairment; however, their specific neuroprotective effects in the context of AD remain unclear (Peng and Zhou, 2024). A range of additional herbal remedies with anti-inflammatory and neuroprotective properties may contribute to reducing neuronal stress and fostering repair mechanisms via the regulation of long non-coding RNAs (lncRNAs) and microRNAs (Li et al., 2024). Herbs like *Ginkgo biloba* and *Anemone altaica* offer distinctive therapeutic benefits. At the same time, ashwagandha is noted as a nerve tonic and antioxidant, potentially enhancing memory and cognitive function by raising acetylcholine levels (Mikulska et al., 2023; Supplementary Table 1).

**TABLE 1:** Neuroprotective properties of selected herbs commonly used in AD treatment to restore and enhance memory and cognitive function.

	Herb	Neuroprotective effect	Bioactive compounds	References
1	*Allium Sativum*	Antioxidant, anti-inflammatory, neuroprotection	Allicin, ajoene, S-allyl-cysteine, diallyl sulfide	(Saini et al., 2021)
2	*Anemone altaica*	Antioxidant, anti-inflammatory, neuroprotection, Aβ degredation	Triterpenoid saponins, ranunculin, anemonin, protoanemonin, flavonoids, phenolic compounds	(Zieneldien et al., 2022)
3	*Centella asiatica*	Reduces oxidative stress, Aβ formation, mitochondiral health, mood memory	Triterpenes, phenolic compounds, rutin, kaempferol, quercetin, gallic acid, luteolin, catechin	(Zieneldien et al., 2022)
4	*Curcuma longa*	Antioxidant, anti-inflammatory, blocks Aβ formation	Curcumin, demethoxycurcumin, bisdemthoxycurcumin, volatile oil	(Kushwah et al., 2023)
5	*Glycyrrhiza uralensis*	Apoptosis, antioxidant, neuroprotective	Trirerpene saponins, flavonoids, licochalcones, pheolic compounds	(Wei et al., 2021)
6	*Hypericum perforatum*	Improve microglial viability, Aβ toxicity, relieve nerve pain, anti-inflammatory	Hyperforin, hypericin, flavonoids, phenolic acids like tannin and xanthone	(El Menuawy et al., 2024)
7	*Panax ginseng*	Antioxidant, anti-inflammatory, neuroprotection, immunomodulation	Ginsenosides, gintonin, pectin, polysaccharides	(Zhang et al., 2023)
8	*Rosmariunus officinalis*	Helps cognitive function, imrpoves memort, prevents damage to nerves, anti-inflammatory, reduces anxiety and stress	Rosmarinic acid, carnosic acid, carnosol, flavonoids	(Chen et al., 2021)
9	*Salvia officinalis*	Improves cognitive functions, pain reliving, improve memory, anti-inflammatory, antioxidant	Flavonoids, phenolic acids, terpenes, rosmarinic acid, ellagic acid, volatile components	(Markova et al., 2022)
10	*Withania somnifera*	Energy, free radical scavenging activity, antioxidant, anti-inflammatory, memory, cognitive function and blocks Aβ formation	Ergostane-type steroidal lactones, phytosterols sitoinodsines VII-X, beta sitosterols, and alkaloids	(Zieneldien et al., 2022)

The bioactive compounds identified and isolated from these medicinal plants include flavonoids, phenolic lignans, tannins, polyphenols, triterpenes, sterols, and alkaloids. Research indicates these phytochemicals exhibit antioxidant, anti-inflammatory, anti-amyloidogenic, anti-tau, and anticholinesterase activities (Chen et al., 2021).

Despite the availability of these treatments, it is important to note that there is currently no cure for AD; existing therapies broadly address symptomatic relief without stopping disease progression. However, future therapies, including immunotherapies that target amyloid plaques, are undergoing clinical trials and show promise for more specific interventions. Additionally, innovative high-throughput multi-omics approaches are making strides in identifying biomarkers that could aid in understanding AD’s pathophysiology and refining therapeutic strategies. These approaches strive to uncover reliable biomarkers associated with the characteristic features of Alzheimer’s, such as Aβ plaques and neurofibrillary tangles, highlighting the significant hurdles in biomarker discovery and disease characterization.

## 5 Multi-omics studies to detect early biomarkers for Alzheimer’s disease

Multi-omics studies that integrate comprehensive molecular data analysis across different stages of the disease, including preclinical, symptomatic, and advanced stages, offer valuable insights into the mechanisms of AD causes and progression while improving diagnostic accuracy. Biomarkers, measurable molecular indicators of disease presence and progression, play a crucial role in this approach. In AD, biomarkers obtained from biofluids such as urine, blood, and plasma can offer important molecular signatures. These signatures aid in early detection, targeted therapies, and ongoing disease monitoring, ultimately contributing to the development of potential treatments.

**FIGURE 1:**
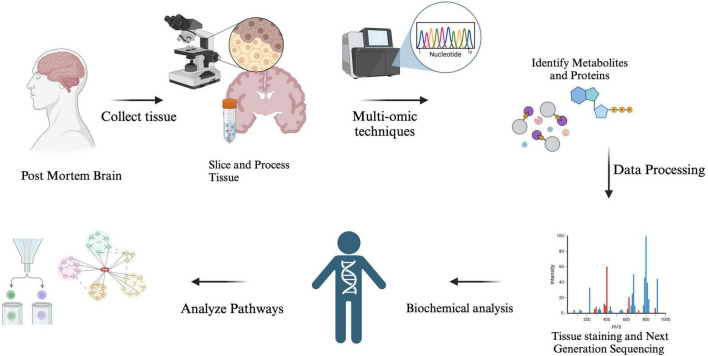
The analysis of Alzheimer’s disease (AD) biomarkers begins with tissue sampling, where brain tissue is collected post-mortem through biopsy. The samples are then prepared, processed, and molecular extraction is performed to isolate proteins, RNA, or metabolites. Researchers utilize techniques such as mass spectrometry and RNA sequencing, combined with computational models, to detect key biomarkers that aid in diagnosis, disease tracking, and the development of potential treatments.

While AD and other common neurodegenerative disorders are characterized by the pathological hallmarks of tangles and plaques, identifying reliable biomarkers has been a significant challenge. Over the years, high-throughput multi-omics-based approaches have been employed to explore dependable biomarkers, creating new opportunities to understand the pathophysiology associated with different molecular states. Multi-omics studies advance our understanding of AD by examining genetic variation, regulatory mechanisms, and epigenetic modifications, exploring a broad spectrum of biological processes to identify biomarkers and elucidate disease pathology. Specifically, genomics, transcriptomics, and Epigenomics provide complementary insights into genetic predisposition, gene expression patterns, and heritable modifications that influence disease progression (Figure 1).

### 5.1 Genomics

The first genetic risk factor identified for AD is the dominant amyloid precursor protein (APP), a type I transmembrane protein cleaved to release Aβ. Thirty mutations in the APP gene on chromosome 21 have been discovered, with twenty-five of these mutations associated with AD and Aβ accumulation (Sirisi et al., 2024). Interestingly, a protective mutation, A673T, has been found to reduce Aβ secretion and lower the risk of developing AD. Alongside APP, other significant genes involved in AD include Presenilin-1 (PSEN1) and Presenilin-2 (PSEN2) (Kim et al., 2024). Mutations in PSEN1 account for around 80% of monogenic AD cases, while PSEN2 mutations are rarer and have a limited effect (Supplementary Table 1). It has been proposed that PSEN1 mutations may hinder neurogenesis by increasing the susceptibility of neural stem cells to amyloid toxicity, potentially leading to cognitive decline (Maksour et al., 2024). These genes influence γ-secretase activity, thereby modulating the ratios of Aβ by elevating Aβ42 levels and decreasing Aβ40 levels; however, the consequences of these alterations in AD continue to be explored (Stanciu et al., 2022). Various factors, including metabolic stress and altered transcription factors, can disrupt cellular homeostasis, further contributing to neuronal injury. Other genes implicated in AD are ATP-binding cassette transporter A1, clusterin, bridging integrator 1, evolutionarily conserved signaling intermediate in the toll pathway, estrogen receptor, and numerous vitamin D receptor gene polymorphisms (Breijyeh and Karaman, 2020). The downregulation of XRCC6, which is crucial for initiating DNA repair, has been observed in neurons characterized by AD. Additionally, age-related DNA damage that occurs during the expression of learning-related genes may accelerate the progression of AD (Lin et al., 2020).

**FIGURE 2:**
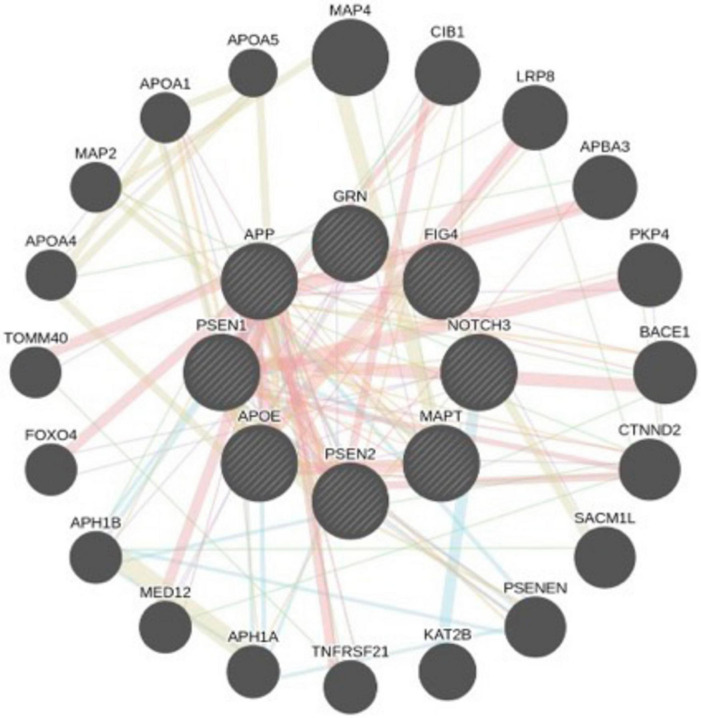
Systems network of AD-associated genes, with key genes highlighted in larger circles. Physical interactions and co-expression patterns are analyzed using a network prediction database. Red lines indicate physical interactions, purple lines represent co-expression, green lines denote genetic interactions, and blue lines signify pathway associations or colocalization within the same organelle. This network visualization offers insights into the complex molecular interplay underlying the pathology of Alzheimer’s disease.

Furthermore, genetic mutations differ based on ancestry, underscoring the importance of population-specific research on AD. Astrocytes in individuals with AD display changes in gene expression, particularly concerning glutamate receptor subunits, which can disrupt molecular pathways and ion balance (Young-Pearse et al., 2023). We present here several variants in individuals associated with AD that have been reported in ClinVar. These variants may also be suitable for application in genome editing technologies (Table 2). Furthermore, a GeneMania interaction network was deciphered between genes responsible for AD (Figure 2).

**TABLE 2:** Characterization of potential genetic markers identified via genomics research associated with AD and dementia risk.

Gene	Mode of action	Description	References
**Genomics**			
ABCA7	Transports fats and cholesterol out of cells and helps clear amyloid-beta.	Supports brain health, mutations increase Alzheimer’s risk.	Li et al., 2021
ADAM10	Cuts APP in a way that prevents harmful amyloid-beta production.	Protects against Alzheimer’s by promoting healthy protein processing.	Kamboh, 2022
ADAM17	Cuts proteins involved in inflammation and cell signaling.	Regulates inflammation and cell communication, linked to Alzheimer’s and cancer.	Kamboh, 2022
APOE	Carries fats and cholesterol in the brain and helps clear away amyloid-beta.	Supports brain health, but a specific version (APOE4) increases Alzheimer’s risk.	Neuner et al., 2020
APP	Gets cut by enzymes (like PSEN1 and BACE1) to produce amyloid-beta, a sticky protein fragment.	Normally helps with brain function, but when cut incorrectly, it forms plaques that cause Alzheimer’s.	Orobets and Karamyshev, 2023
BACE1 (Beta-Secretase 1)	Cuts APP to produce amyloid-beta, the sticky protein that forms plaques.	Plays a key role in Alzheimer’s pathology, a major drug target.	Kamboh, 2022
BIN1 (Bridging Integrator 1)	Helps shape cell membranes and supports waste removal in neurons.	Protects brain cells, linked to Alzheimer’s risk.	Andrade-Guerrero et al., 2023
Cas-9/Cas-12/Cas-13 (CRISPR-associated proteins)	Acts like molecular scissors that cut DNA or RNA at specific locations.	Used in gene editing to fix or modify genes.	Xu and Li, 2020
CD2AP(CD2-Associated Protein)	Helps organize the cell’s internal structure and supports waste clean up.	Maintains cell health, mutations increase Alzheimer’s risk.	Andrade-Guerrero et al., 2023
CD33	Slows down activity of brain’s immune cells	Regulates inflammation, certain variants increase Alzheimer’s risk.	Andrade-Guerrero et al., 2023
CLU	Absorbs up harmful proteins like amyloid-beta.	Protects brain cells, linked to Alzheimer’s risk.	Andrade-Guerrero et al., 2023
CR1 (Complement Receptor 1)	Helps immune cells clear away debris, including amyloid-beta.	Supports brain clean up, variants increase Alzheimer’s risk.	Andrade-Guerrero et al., 2023
KLOTHO	Works like a shield, protecting cells from aging and damage.	Promotes longevity and brain health, low levels are linked to Alzheimer’s	Serrano-Pozo et al., 2021
PICALM	Helps transport materials in and out of cells.	Maintains cell function	Andrade-Guerrero et al., 2023
PSEN1	Acts like a pair of scissors that cuts proteins, including the amyloid precursor protein (APP) to produce smaller fragments.	Helps process proteins in the brain, but when it malfunctions, it can lead to Alzheimer’s disease	Yang et al., 2023
PSEN2	Similar to PSEN1, it cuts proteins like APP into smaller pieces.	Involved in protein processing, mutations can cause early-onset Alzheimer’s.	Gan et al., 2023
SORL1	Guides the amyloid precursor protein (APP) to the right place in the cell to prevent harmful amyloid-beta production.	Protects against Alzheimer’s by reducing amyloid build up.	Li et al., 2021
TREM2	Acts like an antenna on immune cells in the brain, detecting damage and triggering clean up.	Helps microglia clear debris and protect neurons	Li et al., 2021
TREM2	Detects damage and triggers clean up in brain’s immune cells	Helps microglia clear debris and protect neurons	Li et al., 2021
XRCC6	Binds to broken DNA ends and helps glue them back together by recruiting other repair proteins.	Repairs DNA damage and protects cells from harmful mutations	Chen et al., 2024

Over the years, advancements in genome editing have progressed significantly, evolving from zinc finger nucleases and transcription activator-like effector nucleases (TALENs) to clustered regularly interspaced short palindromic repeats (CRISPR) methods (Katam et al., 2022). CRISPR-Cas9, a powerful genome-editing tool that can target a sizable genetic variant, aids in uncovering molecular mechanisms behind neurodegeneration, identifying potential therapeutic targets, and exploring gene-editing strategies that might ultimately prevent or treat AD. Scientists can investigate how specific genetic changes impact AD pathology by introducing or correcting mutations in these genes through cell cultures and animal models (Xu and Li, 2020). These models allow researchers to observe the effects of genetic modifications within a whole organism, which is essential for confirming physiological outcomes and understanding how genetic variations influence AD development *in vivo* (Blanchard et al., 2022).

The Cas system consists of two classes with six subtypes that utilize different Cas proteins depending on the genetic material and system configuration. Cas9 is a single-protein DNA cutter used in gene editing, while Cas12 targets DNA and Cas13 modifies RNA. Cas12 can detect small mutations, including those that affect DNA methylation, whereas Cas13 focuses on RNA modifications in tau proteins, characteristic of AD pathology. Researchers have applied CRISPR-Cas9 to edit genes such as APP, PSEN1, and APOE, contributing to understanding pathways related to AD. The modification of APOE alleles *in vitro*, particularly in neurons and glial cells derived from human stem cells, marks a significant advancement in AD research (Rahimi et al., 2024). Researchers have successfully altered the genetic sequence of the ApoE gene within cultured human neurons and glial cells using CRISPR-Cas9. This development enables the creation of more accurate cellular models of AD that mirror genetic variations found in humans, such as the ApoE4 allele, which is a significant risk factor for developing the disease. However, despite the progress made in human cell culture, the gene-editing technique has not yet been tested in ApoE knock-in mice, which is a crucial next step for advancing research (De Plano et al., 2022).

### 5.2 Transcriptomics

The potential for modifying AD-related gene expression presents exciting therapeutic opportunities. Transcriptomic techniques reveal alterations in gene expression that contribute to AD risk factors, including the upregulation of stress and inflammatory response genes, non-coding RNAs, alternative splicing events, and copy number variants, opening exciting therapeutic opportunities (Bagyinszky et al., 2020). Several differentially expressed genes (DEGs) across various cell types have been identified using the single cell RNA seq, enhancing our understanding of the molecular mechanisms underlying AD (Spurgat and Tang, 2022). Comparative analyses of brain cells from AD patients and age-matched controls emphasize the connection between mitochondrial dysfunction and AD. Notable genes like ZFP36L1, RERE, PURA, OGT, SPCS1, SOD1, and NDUFS5 show consistent expression alterations across 22 brain datasets (Marmolejo-Garza et al., 2022; Table 2). Transcriptomic changes reflect more widespread stress responses at later stages of AD that correlate with increasing levels of brain damage. Single-cell and single-nucleus RNA sequencing (sc/snRNA-seq) techniques have determined differentially expressed mitochondrial genes, highlighting their significant post-transcriptional roles in energy demand regulation. Expression levels of mitochondrial RNA can vary by tissue, reflecting the diverse functions of mitochondria in different cellular environments (Mei et al., 2023). Astrocytes, vital for maintaining brain homeostasis, exhibit significant transcriptomic shifts in AD. There is an upregulation of inflammatory and stress response genes, including CRYAB and GFAP, while glutamate metabolism and synaptic remodeling genes, such as SLC1A2 and SLC1A3, experience downregulation (Saura et al., 2023). Aβ proteins compromise synaptic plasticity and disrupt BDNF-TrkB retrograde signaling pathways. Treatments with Aβ1-42 have led to increased levels of axonal mRNA for AD-related genes like APP, ApoE, and CLU, suggesting their involvement in disease progression (Gao L. et al., 2022; Gao X. et al., 2022). Single-nucleus RNA sequencing has revealed cell-specific changes in gene expression during the early stages of AD, impacting processes like myelination, inflammation, and neuronal survival.

### 5.3 Epigenomics

Advanced technologies, including proteins like CLOuD9 and light-activated dynamic looping (LADL), are used to engineer chromatin for precise gene regulation, highlighting the importance of 3D chromatin organization. CRISPR-GO and live-cell imaging enabled us to study chromatin changes in real-time (Rahman and Roussos, 2024), while epigenetic editing using CRISPR-Cas9 and pharmacological interventions shows promise as a therapeutic approach for AD (Fisher and Torrente, 2024). DNA methylation affects gene expression and chromatin accessibility, ultimately influencing the production of Aβ, calcium homeostasis, and neuronal survival, implicated in oxidative stress and synaptic plasticity (Villa and Combi, 2024). This process links environmental factors, such as homocysteine levels, to the progression of AD (Martinez-Feduchi et al., 2024). Oxidants released by immune cells, particularly from microglia, can alter DNA methylation, further exacerbating neuroinflammation and oxidative stress (Seddon et al., 2024). MicroRNAs, specifically miR-451a and miR-455-3p, play a regulatory role in neurotrophic factors like brain-derived neurotrophic factor (BDNF), neuroinflammation, and neurotransmitter balance, thereby connecting mild behavioral impairments to amyloid/tau pathology (Angelopoulou et al., 2024). Emerging research has identified novel post-translational modifications (PTMs) such as phosphorylation, acetylation, and ubiquitination as potential therapeutic targets (Qin et al., 2024; Table 2). Moreover, the epigenetic regulation of non-coding RNAs affects shared genes such as APOE, BDNF, ACE, FTO, and FNDC5, which are important for muscle mass, mobility, and cognition (Raleigh and Orchard, 2024). BRD4, a critical chromatin remodeling factor, plays a complex role in aging and disease (Sun et al., 2024). Histone acetylation, which is primarily affected in AD, is regulated by histone deacetylases. Inhibitors of these deacetylases can reverse hypoacetylation, improving cognition, memory, and neuroplasticity in preclinical models by promoting neuronal gene transcription and reducing tau and amyloid dysregulation (Pereira et al., 2024).

### 5.4 Proteomics

Recent advancements in mass spectrometry, dimethyl labeling, isobaric tandem mass tags (iTRAQ), and laser capture microdissection have enabled comparisons between symptomatic and asymptomatic Alzheimer’s patients’ brains against those of healthy controls. These innovative methods allow researchers to integrate all cellular proteins, leading to a comprehensive understanding of a system’s biology combined with minimally invasive diagnostic methods. Three commonly used techniques include: 1. cerebrospinal fluid (CSF) collection to assess central nervous system health and neuronal damage; 2. Plasma collection is cost-effective and contains proteins from all body tissues but is complicated by high albumin content that affects protein extraction; 3. Urine collection may contain plasma proteins and potential biomarkers like SPP1, GSN, and IGFBP7 (Jain and Sathe, 2021).

Utilizing proteins as biomarkers and studying their alterations aims to enhance early diagnosis. This breadth of analysis encompasses thousands of proteins involved in energy metabolism, glycolysis, oxidative stress, apoptosis, signal transduction, and synaptic function. However, specific issues arise, such as using polystyrene tubes, which can lead to the loss of “sticky” proteins like Aβ and introduce blood contamination that may degrade proteins, complicating biomarker analysis (Awasthi et al., 2022). Identifying specific biomarkers for Alzheimer’s is made challenging by the presence of overlapping pathologies in many patients. In neural networks, protective proteins include cytoskeleton cross-linking proteins like moesin, ezrin, and radixin, while inflammatory proteins include CAV1, COL6A1, and COL6A3 (Rayaprolu et al., 2021). Other noteworthy proteins in this context are TNF-α and miR-224, which is down-regulated in AD patients, as well as Cystatin C, angiotensin-converting enzyme (ACE), SUMO1, and Chitinase 3-like 1; Table 3). Although proteins such as β2-microglobulin and y-globulinsare associated with AD, they have not yet been validated as potential biomarkers (Mayo et al., 2021). Paraoxonase 1 (PON1) shows promise as a risk factor for AD, mainly due to its anti-inflammatory and anti-apoptotic properties, as low PON1 activity levels correlate with advanced disease.

**TABLE 3:** Characterization of potential genetic markers identified via proteomics research associated with AD pathology.

Protein	Mode of action	Description	Reference
Albumin	Involved in maintaining fluid balance and transportation	Inhibits the formation of Aβ fibrils, disrupts the blood brain barrier	(Kim et al., 2020)
Alpha-1 antitrypsin	Prevents breakdown of lung tissue, regulates the release of white bloodcells	Overexpressed in AD, correlates to disease severity, influences Aβ deposition	(Emwas et al., 2021)
Apo-A1	Plays key roles in lipid metabolism and cardiovascular heatlh	Correlates to disease severity, influences Aβ deposition, inflammation, and oxidative stress	Tong et al., 2022
Beta-2 microglobulin	Helpful in immune system, stabilizes cells on the cell surface	Promotes amyloid plaques, neurotoxicity, brain damage and cognitive decline	(Zhao Y. et al., 2023)
Cathepsin D	Breaks down lysosomal proteins and involved in cell death and response	Breaks down Aβ and tau proteins, a possible sign of response to AD progression	(Suire et al., 2020)
Caveolin 1	Structural functions,involved in endocytosis, signal transductions, and cholesterol homeostasis	Connected to preserving neuronal and synaptic morphology, may prevent neurodegeneration	(Wang et al., 2021)
Chitinase	Inhibits fungal growth, degrades chitin	Breaks down proteins that are toxic to the brain and lead to AD, inflammatory biomarkers	(Connolly et al., 2023)
Chromogranin A	Releases hormones and neurotransmitters from neuroendocrine cells	Indicator of potential synaptic loss, neuronal damage, disease progression	(Quinn et al., 2021)
COL6A1/COL6A3	Maintains structure and function of extracellular matrices	Protects neurons from damage and oxidative stress, impairs autophagy	(Muraoka et al., 2021)
Complement Factor 1 (CF1)	Breaks down activated complement proteins and protects healthy cells	Contributes to acitvation of microglia and astrocytes, progression of Aβ plaques and anlges, protective effects	(Veteleanu et al., 2023)
Cystatin C	Monitors kidney function, produced by all nucleated cells	Associated with cognitive impairment, inhibits Aβ formation, colocalizes with Aβ	(Wang et al., 2023)
Ficolin-2	Triggers immune response, activates complement cascade, regonizes pathogens	Linked to brain atrophy and cognitive decline, clears apoptotic cell debris	(Shi et al., 2021)
Haptoglobin	Defensive role against oxidative stress and inflamation, gets rid of hemoglobin outside red blood cells	Anti-inflammatory properties, binds to Apo-A1, oxidative stress, disease biomarker, structural effects	(Bai et al., 2023)
Nerve growth factor inducible (VGF)	Balances energy, regulates circadian rythmn and is involved in neurite growth and neuroprotection	Correlates to disease severity, inflammation, and oxidative stress	(Quinn et al., 2021)
Neuronal pentraxin-2	Regulates synapse activity and neuroplasticity, simulated excitatory synaptogenesis	Indicator of cognitive impairment, memory decline, and specific excitatory synapses	(Belbin et al., 2020)
Paraoxonase 1	Protects against organophophate poisoning and vascular disease, metabolizes oxidized phospholipids	Associated with cognitive impairment, oxidative stress, and biomarkers for AD	(Perła-Kaján et al., 2021)
Secernin-1	Regulator of exocyosis in mast cells, involved in synaptic vesicle recycling	Colocalization of neurofibrillary tangles, increased phosphorylated tau binding, accumulation of Aβ plaques	(Weiner et al., 2023)
Secretogranin-2	Packages hormones and neuropeptides into secretory granules	Indicator of potential synaptic loss, neuronal damage, disease progression	(Quinn et al., 2021)
SUMO1 (small ubiquitin-like modifier 1)	Involved in nuclear transport, DNA replication and protein stability	Promotes tau aggregation, impacts phosphorylation, colocalizes with interneuronal tau	(Takamura et al., 2022)
Superoxide dismutase	Plays important role in antioxidant defense and maintains cellular health	Protects against oxidative damage and free radicals, can buildup neurotoxic proteins in AD	(Balendra and Singh, 2021)

In contrast, areas like the sensory cortex, motor cortex, and cerebellum show less impact (Marsillach et al., 2020). Challenges arise from small sample sizes that complicate the detection of protein differences across multiple comparisons. In contrast, larger sample sizes may yield too many variations, making it difficult to establish consistency.

### 5.5 Metabolomics and lipid-omics

Over the past decade, metabolomics and lipidomics have made significant advances in identifying critical changes in metabolites that affect mitochondrial function, neuroinflammation, and cognitive decline. Lipidomics focuses explicitly on the role of lipid metabolism in neuronal health and amyloid pathology. Genetic predisposition to diseases and traits can be quantified through polygenic scores derived from metabolomic data and multi-omics approaches (Joshi et al., 2023). Various metabolomic platforms are available, each with advantages and challenges, especially concerning sensitivity to external variables, reproducibility, and costs (Chen et al., 2022). Targeted metabolomics focuses on specific metabolites based on hypotheses, while untargeted methods enable broad profiling of numerous metabolites within a sample (Trifonova et al., 2023). The early applications of metabolomics in AD began in 2009 with gas chromatography-mass spectrometry and linear ion trap (LTQ) orbit trap technologies. However, challenges such as variability and small sample sizes prevented the discovery of statistically significant biomarkers (Reveglia et al., 2021).

Later research utilizing ultra-performance liquid chromatography (UPLC) with a hybrid quadrupole time-of-flight (Q-TOF mass spectrometer) and gas chromatography time-of-flight mass spectrometry (GC-TOF-MS) platforms analyzed participants with AD, mild cognitive impairment, and healthy controls, identifying distinct metabolites like arachidonic acid, N, N-dimethylglycine, and thymine that differentiate AD patients (Yin et al., 2023). Furthermore, decreased levels of oleamide, histidine, monoglycerides, and increased phenylacetylglutamine were noted in AD patients, suggesting potential inflammatory responses (Table 4). Further analyses indicated elevated levels of cortisol and cysteine and reduced uridine in patients. Thymidine and uracil are crucial in nucleic acid metabolism, influencing mitochondrial function. Taurine, a key amino acid in the central nervous system, has been proposed to enhance cognitive function and protect against memory loss without impairing motor skills (Ramírez-Guerrero et al., 2022). Several substances with neuroprotective properties, such as kurarinone, tauroursodeoxycholic acid (TUDCA), and curcumin, have shown promise in enhancing motor behavior and reducing neuroinflammation. However, these treatments have yet to yield specific biomarkers for assessing human neuroprotection (Franco et al., 2024). Metabolomic changes linked to AD include fluctuations in phospholipids, amino acids, and other metabolites and altered kynurenine pathways. Metabolic profiling has revealed elevated levels of alanine, glutamate, and glycerophosphocholine, while decreased lactate and N-acetyl aspartate have been reported in AD patients, indicating a potential signature for the disease (Vignoli and Tenori, 2023). Research regarding insulin resistance has identified glucose and fructose as key metabolic biomarkers, while heightened ceramide levels have been associated with mitochondrial dysfunction and inflammation in AD patients (Amin et al., 2023). Notably, differences have been observed between genders, such as a lower D-serine ratio in women with AD compared to men (Yin et al., 2023). D-serine binds to receptors, activating the N-methyl-D-aspartate receptor (NMDAR) and mediating excitotoxicity. Studies indicate that D-serine contributes to excitatory neuronal damage in the hippocampus, influences neuroinflammation, and affects amino acid balance. Reducing D-serine levels in mouse models has decreased hippocampal neuronal death and neuroinflammation, presenting a viable NMDAR-based treatment strategy for AD (Ni et al., 2023). Nevertheless, a significant challenge in metabolomics is the inconsistency of results due to variability in metabolite biomarkers based on sample sources. Despite identifying numerous potential biomarkers, the statistical robustness is often limited due to challenges in controlling external influences (Batra et al., 2024).

**TABLE 4:** Exploring genetic markers associated with Alzheimer’s disease and dementia risk through metabolomics and lipidomics research.

Metabolite	Mode of action	Description	References
Acetate	Inhibts nuclear factor-kB to reduce levels of COX-2 and IL-1β through GPR41	Regulates microglia maturation, fuction, and disease progression	(Erny et al., 2021)
Adensoine	A depressant in the central nervous system and regulated the immune system, produced by ATP dephosphorylation	Over activation of adenosine A2A recptors are linked to cognitive impairment	(Ji et al., 2023)
Alanine	Converted to pyruvate which Is an important energy substrate for brain production of neurons	A deficit in alanine metabolism leads to oxidative stress and mitochondiral dysfunction	(Polis and Samson, 2019)
2-aminoadipic acid	A metabolite in the lysine breakdown pathway	A marker for protein oxidative stress and is increased in the blood brain barrier in AD	(Mao et al., 2023)
Arachidonic acid	Converted by cyclooxygenases-1/2 and prostaglandin sythases into PGE2 and PGD2	Contributes to occurrence and progression of neuroinflammation	(Xia et al., 2024)
Aspartate	An excitatory neurotransmitter in the brain with importance in neural communication	Decreased in AD patients and contributes to the disruption in metabolism	(Chang et al., 2024)
Betaine	Oxidized by choline in the mitochondria and cytosol to helo regulate stress and fluids	May reduce Aβ plaques, tau phosphorylation and oxidative stress	(Alipourfard et al., 2023)
Creatine	Is converted to phosphocreative and is an energy supply to neurons	Creatine deposits in AD patients indicate abnormal metabolism and cellular damage	(Smith et al., 2023)
Citicoline	A component of citicoline which makes acetylcholine and cell membranes +B10	Helps with memory and increases brain uptake of choline	(Piamonte et al., 2020)
N, N-dimethylglycine (DMG)	Is produced when the body metabolized choline in to glycine	A blood-based biomarker and improves memory and cognitive impairment	(Varesi et al., 2022)
Glutamine	Is released from astrocytes and activates extrasynaptic NMDARs triggering pro-apoptic signaling and synaptic damage	Damages nerons and causes memory loss	(Yu et al., 2023)
Histidine	An important metabolite and precursor to carosine, an anti-inflammatory, anti-oxidant and neurotransmitter	An equillibrium between tautomers that are likely to form β sheets and contribute to AD	(Salimi et al., 2022)
Lysine	Lysine degradation forms by saccharopine pathways in the mitochondria	A factor in neurotoxcity through ubiquitination that occurs on tau proteins leading to tau tangles	(Persico et al., 2022)
Mannose	Decrease in high-mannose disrupts the glycoslation process	Supports the accumulation of Aβ plaques in the brain	(Tang et al., 2023)
Myoinositol	A precursor of the phosphatidylinositol second messenger system	Associated with changes in mood state	(Voevodskaya et al., 2019)
Phosphocholine	Affects the brain’s cell membranes and cholinergic neurons	Decreased in the brain and plasma of AD patients showing signs of cell damage	(Shanks et al., 2022)
Thiamin	A key factor in glucose metabolism	Deficient in AD patients and linked to cognitive decline	(Fessel, 2021)
Threonine	Threonine metabolites impact cellular energy production and neurodegeneration	Leads to abnormal tau phosphorylation and neurofibrillary tangles, increases oxidative stress	(Zhao H. et al., 2023)

Altered lipid metabolism plays a significant role in aging, increasing plasma triglyceride and lipoprotein levels while decreasing triglyceride clearance and lipoprotein lipase activity. Peroxisomal disorders, which result in inherited ether lipid deficiencies, have been linked to AD (Hussain et al., 2019). These metabolic changes affect lipid transport and biochemical pathways across various organs (Chung, 2021). Mitochondria are crucial for lipid metabolism, yet their functionality declines with age, emphasizing the importance of metabolomics and lipidomics in AD research (Yin et al., 2023). The lipid metabolites and pathways strategy, or Lipid MAPS, categorizes lipids into eight groups: fatty acids, glycolipids, glycerophospholipids, sphingolipids, sterols, phenols, saccharolipids, and polyketides, each identified uniquely (Meikle et al., 2021). The brain, abundant in glycerophospholipids and sphingolipids, relies on these lipids for structural integrity and functionality. Moreover, decreased levels of plasmalogens have been observed with aging and AD; however, the results of replenishment treatments are inconsistent. Lipids are essential for numerous cellular processes, including signaling, maintaining membrane structure, and biological messaging. Although findings remain inconclusive, an upregulation of brain cholesterol synthesis in AD has been observed, and cholesterol accumulation in senile plaques suggests its potential role in the disease’s pathophysiology (Yin et al., 2023).

## 6 Advances in diagnostics and personalized medicine

**FIGURE 3:**
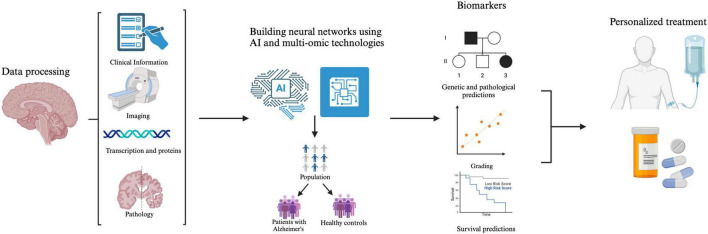
The integration of multi-omics data, facilitated by artificial intelligence (AI), provides a more comprehensive understanding of AD pathology. By analyzing this data, models can identify dysregulated pathways and various biomarkers, improving patients’ lives through early diagnosis, risk assessments, and targeted therapeutic interventions.

Advancements in radiomics, neuroimaging, and machine learning are transforming the early diagnosis and management of AD. By integrating brain imaging techniques with AI-driven models, researchers can enhance the accuracy of disease prediction, monitor progression, and personalize medicine. Radiomic analyses provide insight into the structural and functional changes in the brain. Meanwhile, machine learning algorithms examine large datasets to identify biomarkers and predict disease trajectories (Figure 3).

### 6.1 Radiomics and neuroimaging

Brain imaging and genomics are key components in systematically analyzing AD, integrating image preprocessing, region of interest identification, model building, genomic data extraction, and downstream analysis. Together, these methodologies contribute to the precision medicine approach for AD imaging biomarkers with genomic implications (Li and Luo, 2022). Radionics-based analysis and nuclear medicine tools, such as fluorodeoxyglucose (FDG), β-amyloid positron emission topography (PET), and dopamine transporter single proton emission computed tomography (SPECT), combined with advanced computer technology, can enhance classification and prediction rates for AD. The primary goal is the early diagnosis of mild to moderate cognitive impairment and monitoring its progression toward Alzheimer’s as the brain ages. Current studies utilize regions of interest-based radionics and support vector machine classifiers on PET imaging to assess decision-making accuracy in models describing AD’s stage and severity (Seo et al., 2022). Radiomic network modeling targeting the cerebellum shows promise for early identification of the preclinical stage of AD. These integrated models outperform traditional hippocampal models in patients with mild cognitive impairment, effectively predicting distinct risks associated with the progression of amyloid and tau pathologies (Chen et al., 2025). For example, models using 18F-FDG-PET images interpret deep-learning radionics to understand better and predict the pathway from mild impairment to AD. Techniques like Extreme Gradient Boosting (XGBoost) illustrate the focus areas within the model, while methods such as gradient-weighted Class Activation Mapping (Grad-CAM) and Shapley Additive exPlanations (SHAP) are crucial for identifying factors that influence predictions (Jiang et al., 2024).

### 6.2 Machine learning

Integrating AI with radiomics and brain imaging shows significant potential to enhance personalized medicine and clinical treatments for AD. Machine learning techniques, such as logistic regression and convolutional neural networks, are employed to develop diagnostic models that analyze various brain images for signs of AD (Bevilacqua et al., 2023). By utilizing AI to monitor and predict the formation of tangles and plaques, the approach to treating early-onset AD patients could be fundamentally transformed. GNNexplainer technology aids in identifying key variables and genetic pathways that play a crucial role in understanding treatment outcomes and success in clinical trials focused on AD (Aghakhanyan et al., 2023). Current research methodologies leverage mathematical models and computational techniques to simultaneously examine DNA and brain alterations. The amyloid-tau-neurodegeneration (ATN) framework, which encompasses amyloid, tau, and neurodegeneration, helps delineate the accumulation of these components and the resulting implications for brain function (Li et al., 2022). While a significant amount of AI research in dementia relies on the comprehensive Alzheimer’s Disease Neuroimaging Initiative (ADNI) dataset, which is praised for its size and accessibility, it has limitations, such as the underrepresentation of non-Alzheimer’s dementias and inherent biases, indicating the need for broader recruitment from memory clinics and the systematic collection of longitudinal data (Winchester et al., 2023). Emerging studies indicate that AI-driven evaluations of blood-based biomarkers and cerebrospinal fluid (CSF) markers, including plasma p-tau, the Aβ42/40 ratio, and neurofilament light (NfL), facilitate early detection of Alzheimer’s, presenting a less invasive alternative to traditional CSF tests and PET scans (Chatterjee et al., 2022). Advances in AI-powered genomic analysis, incorporating deep learning models and polygenic risk scores, enhance the identification of genetic variants linked to Alzheimer’s, allowing for more accurate risk assessments and personalized therapeutic strategies (Zhou et al., 2023). Machine learning enhances the creation of individualized treatment plans by analyzing diverse patient data, such as genetic profiles, brain scans, and medical histories, to identify patterns and predict treatment responses. These systems utilize various learning methods to analyze data, uncover new subtypes of AD, and interpret intricate datasets like brain images. By continuously tracking the patient’s condition and modifying treatment accordingly, AI aims to deliver more personalized care, increasing treatment effectiveness while minimizing side effects (Zhang et al., 2023). The rising interest in these studies among scientists underscores the need for advancements in AI models, particularly regarding how they incorporate biological networks and complex systems, including multi-omics approaches for enhanced pathway analysis. There is also a need to improve the generalizability and reproducibility of results to ensure statistical accuracy. However, despite these promising developments, these AI models are not yet ready for clinical application, as imaging alone cannot reliably diagnose AD and cognitive impairments in aging populations. The synergistic application of multi-omics and emerging brain imaging technologies holds immense potential to revolutionize the treatment landscape for patients with AD.

## 7 Limitations and current challenges

Current limitations on the pathology and even cure of Alzheimer’s disease (AD) are mainly due to a lack of consistent information and ethical testing. Data obtained from postmortem brains cannot provide the same educational value as insights from live brains. Although animal models aid scientists in understanding the disease, they fail to accurately replicate the symptoms of AD as they present in humans. This underlines the significance of leveraging advanced technologies to study and compare healthy aged brains with both early and late-onset Alzheimer’s patients, emphasizing the need for new research to transition into clinical trials promptly. With the genome predominantly transcribed in eukaryotes and non-coding elements playing substantial regulatory roles, there remains a limited understanding of how long non-coding RNAs (lncRNAs) influence neurodegenerative disease modulation. Recent advancements in next-generation sequencing (NGS) technology have led to the discovery of numerous lncRNAs, which warrant further investigation to clarify their regulatory functions and to enhance non-functional activity. Studies have also revealed differential methylation of microRNA (miRNA) and lncRNA genes in human hippocampal tissues affected by epilepsy, illustrating that lncRNAs participate in regulatory pathways related to inflammation and neuronal differentiation in the epileptic brain. This suggests that the differential methylation status of non-coding RNAs is crucial in the pathogenesis of neurodegenerative diseases. Given that lncRNAs affect neighboring genes, it would be intriguing to examine if mutations in these genes are associated with lncRNA modulation. If such relationships exist, they could lead to further inquiry into whether lncRNA sequences function as protospacer adjacent motifs (PAM) within the genome. A wiki-based web resource or tool may serve as an interface to predict interactions between protein-coding genes and lncRNAs. However, while the CRISPR/Cas9 system can delete lncRNA genes associated with AD, identifying functional regions and potential off-target effects from such interventions targeting the complex genomic architecture remains uncertain.

## 8 Conclusion

Alzheimer’s disease (AD) presents a significant global health challenge due to its rising prevalence and the absence of effective treatments. Integrating machine learning, artificial intelligence, and multi-omics technologies holds immense potential to transform AD care, allowing for minimally invasive, efficient, and more precise interventions. Nonetheless, numerous challenges persist, including the generation of vast datasets, variability among samples, divergent interpretations of biological functions, and the intricate characteristics of clinical phenotypes. Machine learning and artificial intelligence (AI) offer advanced tools to decipher complex datasets and uncover hidden patterns. However, a multidisciplinary approach and diverse cohorts characterized by detailed phenotyping are essential to improve precision medicine for dementia. Ongoing research aims to identify consistent omics signatures distinguishing between dementia subtypes, enhance biomarker development through high-dimensional data utilization, and elucidate missing heritability factors in genetic investigations. Understanding the interplay between genetics and dementia risk factors alongside associated biological processes is vital. Efficient techniques for detecting preclinical and prodromal dementia are critical for effective secondary prevention strategies. Recent advancements in blood-based biomarkers have made widespread monitoring feasible, and preventative initiatives are being tailored using digital tools for remote cognitive and behavioral tracking. Researchers could develop precision preventive methods that align risk reduction with optimal therapeutic allocation by establishing at-risk cohorts based on cardiovascular and genetic risk factors interactions.
