# Supplementary material for: Advancements in multi-omics research to address challenges in Alzheimer’s disease: a systems biology approach utilizing molecular biomarkers and innovative strategies
Source: Front Aging Neurosci. 2025 Jul 23;17:1591796. doi: 10.3389/fnagi.2025.1591796 (PMC12325291; doi:10.3389/fnagi.2025.1591796)
Supplement: Supplementary file 2 [file Table_2.docx]

Supplement Table 2: List of genes associated with Alzheimer’s disease along with their associated phenotypes.

| **Gene(s)** | **Protein change** | **Condition(s)** | **Chromosome** | **POs** | **dbSNP ID** | **Molecular consequence** |
| --- | --- | --- | --- | --- | --- | --- |
| PSEN2 | N141I | not provided Alzheimer disease 4 | 1 | 226885603 | rs63750215 | missense variant |
| PSEN2 | M239V | Alzheimer disease 4 | 1 | 226888977 | rs28936379 | missense variant |
| HFE\|HFE-AS1 | H63D, H40D | Familial porphyria cutanea tarda\| Variegate porphyria\| Hemochromatosis type 1\|Transferrin serum level quantitative trait locus 2\|Microvascular complications of diabetes, susceptibility to, 7\|Alzheimer disease Variegate porphyria\| Cardiomyopathy\| not provided\| Hemochromatosis type 1\|Hereditary hemochromatosis\| See cases | 6 | 26090951 | rs1799945 | non-coding transcript variant\| missense variant\| intron variant |
| FIG4 | Q823* | not provided\| Charcot-Marie-Tooth disease type 4\|Yunis-Varon syndrome\| Charcot-Marie-Tooth disease type 4J | 6 | 109796772 | rs745790694 | nonsense |
| PSEN1 | A79V, A75V | Acne inversa, familial, 3\|Pick disease\| Alzheimer disease 3\|Frontotemporal dementia \|not provided | 14 | 73170945 | rs63749824 | missense variant |
| PSEN1 | T112N, T116N | Alzheimer disease 3\|Frontotemporal dementia \|Pick disease\| Acne inversa, familial, 3 | 14 | 73173574 | rs63750730 | missense variant |
| PSEN1 | N135S, N131S | Alzheimer disease 3\|Pick disease Frontotemporal dementia Acne inversa, familial, 3\|Alzheimer disease 3\|not provided | 14 | 73173631 | rs63751278 | missense variant |
| PSEN1 | M139V, M135V | not provided Alzheimer disease 3\|Acne inversa, familial, 3\|Pick disease Frontotemporal dementia Alzheimer disease 3 | 14 | 73173642 | rs63751037 | missense variant |
| PSEN1 | I143T, I139T | Alzheimer disease 3\|Pick disease Frontotemporal dementia Acne inversa, familial, 3\|not provided Alzheimer disease 3 | 14 | 73173655 | rs63750004 | missense variant |
| PSEN1 | M146V, M142V | Alzheimer disease 3 | 14 | 73173663 | rs63750306 | missense variant |
| PSEN1 | M146L, M142L | not provided Acne inversa, familial, 3\|Pick disease Alzheimer disease 3\|Frontotemporal dementia Alzheimer disease 3 | 14 | 73173663 | rs63750306 | missense variant |
| PSEN1 | M146I, M142I | Alzheimer disease 3\|Pick disease Frontotemporal dementia Acne inversa, familial, 3\|not provided | 14 | 73173665 | rs63750391 | missense variant |
| PSEN1 | H163Y, H159Y | Alzheimer disease 3 | 14 | 73186859 | rs63749885 | missense variant |
| PSEN1 | H163R, H159R | PSEN1-related disorder Acne inversa, familial, 3\|Pick disease Alzheimer disease 3\|Frontotemporal dementia not provided Acne inversa, familial, 3\|Dilated cardiomyopathy 1U\|Pick disease Alzheimer disease 3\|Frontotemporal dementia | 14 | 73186860 | rs63750590 | missense variant |
| PSEN1 | G206A, G202A | not provided Pick disease Acne inversa, familial, 3\|Alzheimer disease 3\|Frontotemporal dementia Pick disease Acne inversa, familial, 3\|Alzheimer disease 3\|Frontotemporal dementia Dilated cardiomyopathy 1U\|Alzheimer disease 3 | 14 | 73192712 | rs63750082 | missense variant |
| PSEN1 | I245L, I249L | not provided Acne inversa, familial, 3\|Alzheimer disease 3\|Pick disease Frontotemporal dementia | 14 | 73192840 | rs1362575880 | missense variant |
| PSEN1 | R269H, R265H | Frontotemporal dementia Alzheimer disease 3\|Acne inversa, familial, 3\|Pick disease not provided | 14 | 73198067 | rs63750900 | missense variant |
| PSEN1 | L271V, L267V | Acne inversa, familial, 3\|Pick disease Frontotemporal dementia Alzheimer disease 3\|Alzheimer disease 3 | 14 | 73198072 | rs63750886 | missense variant |
| PSEN1 | E280G, E276G | not provided\| Alzheimer disease 3\|Acne inversa, familial, 3\|Pick disease Frontotemporal dementia Alzheimer disease 3 | 14 | 73198100 | rs63750231 | missense variant |
| PSEN1 | E280A, E276A | Acne inversa, familial, 3\|Pick disease Frontotemporal dementia\| Alzheimer disease 3 | 14 | 73198100 | rs63750231 | missense variant |
| PSEN1 |  | Alzheimer disease 3\|Alzheimer disease Frontotemporal dementia Pick disease Acne inversa, familial, 3\|Alzheimer disease 3 | 14 | 73206385 | rs63750219 | splice acceptor variant |
| PSEN1 | C410Y, C406Y | Acne inversa, familial, 3\|Pick disease Alzheimer disease 3\|Frontotemporal dementia | 14 | 73217225 | rs661 | missense variant |
| PSEN1 | A431E, A427E | Acne inversa, familial, 3\|Frontotemporal dementia Alzheimer disease 3\|Pick disease Alzheimer disease 3\|not provided | 14 | 73219177 | rs63750083 | missense variant |
| PSEN1 |  | Alzheimer disease 3\|Frontotemporal dementia Pick disease\| Acne inversa, familial, 3 |  |  |  |  |
| GRN | R198fs | not provided\| GRN-related frontotemporal lobar degeneration with Tdp43 inclusions Neuronal ceroid lipofuscinosis 11 | 17 | 44350571 - 44350572 | rs1555611136 | frameshift variant |
| MAPT | S352L, S669L, S263L, S292L, S321L, S323L, S294L, S687L, S647L, S744L | Supranuclear palsy, progressive, 1 | 17 | 46018675 | rs63750425 | missense variant\| non-coding transcript variant intron variant |
| MAPT | R406W, R723W, R375W, R317W, R346W, R741W, R348W, R377W, R701W, R798W | not provided Frontotemporal dementia Pick disease Parkinson disease, late-onset\| Progressive supranuclear palsy-parkinsonism syndrome Frontotemporal dementia\| Supranuclear palsy, progressive, 1\|Progressive supranuclear palsy-parkinsonism syndrome | 17 | 46024061 | rs63750424 | missense variant\| non-coding transcript variant\| intron variant |
| NOTCH3 | R578C | not provided Cerebral arteriopathy, autosomal dominant, with subcortical infarcts and leukoencephalopathy, type 1 | 19 | 15187213 | rs769773673 | missense variant |
| APOE | C130R, C156R, L46P, L72P | Familial hypercholesterolemia\|APOE4(-)-FREIBURG | 19\|19 | 44908684 | rs429358\|rs769452 | missense variant |
| APOE | C130R, C156R, R269G, R295G | Familial type 3 hyperlipoproteinemia | 19\|19 | 44908684 | rs429358\|rs267606661 | missense variant |
| APP | V717F, V624F, V661F, V693F, V586F, V607F, V642F, V680F, V698F, V699F | Alzheimer disease not provided\| Alzheimer disease type 1 | 21 | 25891784 | rs63750264 | missense variant |
| APP | V717I, V607I, V624I, V661I, V642I, V680I, V693I, V698I, V699I, V586I | Alzheimer disease not provided Cerebral amyloid angiopathy, APP-related\| Alzheimer disease type 1\|Alzheimer disease type 1\|Cerebral amyloid angiopathy, APP-related | 21 | 25891784 | rs63750264 | missense variant |
| APP | I716T, I623T, I585T, I660T, I641T, I692T, I697T, I698T, I606T, I679T | Alzheimer disease | 21 | 25891786 | rs63750851 | missense variant |
| APP | I606V, I692V, I697V, I698V, I585V, I660V, I641V, I679V, I623V, I716V | Alzheimer disease | 21 | 25891787 - 25891788 | rs2146237965 | missense variant |
| APP | V715M, V622M, V640M, V678M, V584M, V691M, V697M, V605M, V659M, V696M | Alzheimer disease type 1\|not provided Alzheimer disease | 21 | 25891790 | rs63750734 | missense variant |
| APP | T714A, T604A, T639A, T658A, T583A, T696A, T677A, T690A, T621A, T695A | Alzheimer disease type 1\|Alzheimer disease | 21 | 25891793 | rs63750643 | missense variant |
| APP | D563N, D584N, D601N, D619N, D638N, D657N, D670N, D675N, D676N, D694N | Alzheimer disease not provided | 21 | 25891853 | rs63749810 | missense variant |
| APP | E693G, E562G, E669G, E618G, E583G, E637G, E656G, E600G, E674G, E675G | Abeta amyloidosis, Arctic type\| Alzheimer disease type 1 | 21 | 25891855 | rs63751039 | missense variant |
| APP | E693Q, E618Q, E637Q, E656Q, E562Q, E583Q, E669Q, E600Q, E674Q, E675Q | Cerebral amyloid angiopathy, APP-related\| Alzheimer disease | 21 | 25891856 | rs63750579 | missense variant |
| APP | A692G, A655G, A561G, A582G, A617G, A636G, A599G, A668G, A673G, A674G | ABetaA21G amyloidosis Alzheimer disease type 1 | 21 | 25891858 | rs63750671 | missense variant |
| APP | A673V, A654V, A542V, A598V, A580V, A617V, A655V, A563V, A636V, A649V | Alzheimer disease | 21 | 25897619 | rs193922916 | missense variant |
| APP |  | APP-related disorder Alzheimer disease | 21 | 25897626 - 25897627 | rs281865161 | missense variant |
| APP\|GABPA |  | Alzheimer disease |  |  |  |  |
| APP |  | not provided |  |  |  |  |
| APP |  | Alzheimer disease |  |  |  |  |
| ADAMTS1\|  ADAMTS5\|  APP\|ATP5PF\|  CYYR1\|  GABPA\|  JAM2\|MIR155\|  MRPL39 |  | Alzheimer disease |  |  |  |  |
| SAMSN1\|  ADAMTS1\|TMPRSS15\|CYYR1\|GABPA\|ADAMTS5\|APP\|HSPA13\|JAM2\|LIPI\|MIR125B2\|MIR155\|BTG3\|C21orf91\|MIR99A\|ATP5PF\|CHODL\|CXADR\|USP25\|MIRLET7C\|MRPL39\|NCAM2\|NRIP1\|POTED\|RBM11 |  | Alzheimer disease Cerebral amyloid angiopathy, APP-related |  |  |  |  |

The GRCh38 Chromosome and positions are inserted along with Rs ids and molecular consequences
